# 老年套细胞淋巴瘤诊断与治疗中国专家共识（2026年版）

**DOI:** 10.3760/cma.j.cn121090-20260207-00084

**Published:** 2026-05

**Authors:** 中华医学会血液学分会淋巴细胞疾病学组 中国医药教育协会淋巴疾病专业委员会

## Abstract

套细胞淋巴瘤（mantle cell lymphoma, MCL）是非霍奇金淋巴瘤的少见类型，多见于老年人，随着我国人口逐渐老龄化，老年患者数量上升。新型靶向药物和治疗新策略的出现改变了MCL现有的治疗模式和预后，但老年患者耐受性差，需根据综合老年评估制定个体化的治疗方案。因此，中华医学会血液学分会淋巴细胞疾病学组和中国医药教育协会淋巴疾病专业委员会组织相关专家制定了本共识，旨在提高我国临床医师对老年MCL的诊断和治疗水平。

## Abstract (en)

Mantle cell lymphoma（MCL）is a rare type of non-Hodgkin lymphoma that predominantly occurs in elderly patients. In China, the proportion of elderly patients with MCL is increasing with the aging of the general population. The emergence of new targeted drugs and novel approaches has changed the treatment patterns and prognosis of MCL. Meanwhile, due to poor tolerance, elderly patients require individualized treatment strategies based on a comprehensive geriatric assessment. Therefore, the Lymphoid Disease Group of the Chinese Society of Hematology, Chinese Medical Association, and the Lymphatic Disease Society of the Chinese Medical Education Association convened relevant experts to formulate this consensus, to improve the diagnosis and treatment levels of elderly patients with MCL in China.

套细胞淋巴瘤（mantle cell lymphoma，MCL）是非霍奇金淋巴瘤的少见类型，具有独特的组织学、免疫表型及细胞遗传学特征，兼具侵袭性淋巴瘤的侵袭性和惰性淋巴瘤不可治愈的特点。在西方国家，MCL占非霍奇金淋巴瘤（non-Hodgkin lymphoma，NHL）的3％～10％[1]，我国MCL占NHL的1.0％～3.1％[2]–[4]；男女比例为（2～4）∶1；西方国家MCL的中位发病年龄为68岁，我国约为60岁。2021年中国开始进入深度老龄化社会，≥65岁人口占比超14％，2022年、2023年≥65岁人口占比分别为14.9％和15.4％。根据《中国人口预测报告2023版》，中国在2032年左右将进入≥65岁人口占比超过20％的超级老龄化社会，之后持续快速上升，2060年该占比约为40.17％[5]，据此推测我国老年MCL患者将会逐渐增加，需重点关注。

近年来出现了很多治疗MCL的新药，但针对老年MCL的研究较少，缺乏标准的治疗方案。临床采用简化的MCL国际预后评分系统（MCL international prognostic score，MIPI）及加入Ki-67指数的改良MIPI（MIPI-c）进行预后分层，其中年龄是影响预后的因素之一，年龄每增加1岁，患者的生存相对风险增加1.04[6]，年龄越大，预后越差。因此，老年MCL需结合合并症、化疗耐受性等进行综合评估，制定个体化治疗策略。

为进一步提高对老年MCL的认识，规范诊治水平，中华医学会血液学分会淋巴细胞疾病学组和中国医药教育协会淋巴疾病专业委员会组织多学科专家，根据国际及国内相关指南及循证医学证据，结合我国的诊治水平和现状，讨论并制定本共识。本共识已在国际实践指南注册与透明化平台（Practice guideline REgistration for transPAREncy，PREPARE）注册（注册号：PREPARE-2025CN1217）。

一、老年的定义

“老年”的定义在不同领域和社会背景下可能有所差异，并没有固定的年龄界限，在淋巴瘤患者中针对老年的定义也不完全统一，考虑到中国MCL患者的发病年龄特征，结合既往相关临床及转化研究资料[7]–[10]，本共识将年龄≥60岁的患者定义为老年MCL患者。

二、MCL患者的评估

老年患者需要进行必要的体格检查、实验室检查，特别应进行综合老年评估。

1. 体格检查：注意全身浅表淋巴结、肝脾是否肿大及结外器官受累情况。

2. 针对患者的美国东部肿瘤协作组（ECOG）体能状态评分引入老年综合评估（comprehensive geriatric assessment，CGA）。

老年MCL患者面临认知功能下降、伴多种合并症、体能衰弱及营养不良等风险，导致抗肿瘤治疗的不良反应增加；同时老年患者治疗依从性差。为优化治疗策略，引入CGA指导临床决策，不适合化疗或脆弱（unfit/frail）患者需采取低强度治疗方案减少并发症的发生。CGA评估包括认知功能、情绪、营养状况、日常生活活动能力（activities of daily living，ADL）、工具性日常生活活动能力（instrumental activities of daily living，IADL）、多种药物合用（使用五种或更多药物）、客观测量的身体状态（如步态速度、握力或平衡测试）和社会支持等，目前存在多种评估体系。由于MCL缺乏CGA数据，本共识建议参考老年弥漫大B细胞淋巴瘤的简化老年综合评估（sGA），具体分组依据见表1。

**表1:** 简化老年综合评估（sGA）体系的分组标准

指标	sGA分层			
	适合化疗组	不适合化疗组1	不适合化疗组2	脆弱组
ADL（分）	≥5	<5	6	<6
IADL（分）	≥6	<6	8	<8
MCIRS-G	无3～4级合并症 （2级合并症≤8个）	≥1个3～4级合并症 （2级合并症>8个）	无3～4级合并症 （2级合并症<5个）	≥1个3～4级合并症 （2级合并症≥5个）
年龄（岁）	<80	<80	≥80	≥80

**注** ADL：日常生活活动能力；IADL：工具性日常生活活动能力；MCIRS-G：改良老年疾病累计评分表

3. 实验室检查：血常规，生化常规，血清LDH水平，β_2_-微球蛋白水平，乙型肝炎病毒、丙型肝炎病毒和HIV等病毒相关检测及骨髓活检、细胞形态学和流式细胞术检查。

4. 影像学检查：推荐全身PET/CT检查协助发现隐匿性病灶或进行颈、胸、腹、盆腔的增强CT检查。30％～40％的MCL患者在基线时存在胃肠道受累，当怀疑胃肠道受累时应进行胃肠镜检查。建议在考虑应用含蒽环类药物的化疗方案或布鲁顿酪氨酸激酶（BTK）抑制剂（BTKi）治疗前进行心脏功能的评估。若怀疑中枢神经系统受累，建议进行腰椎穿刺和头颅MRI检查。

5. 新一代高通量测序（next-generation sequencing，NGS）检测：MCL异质性高，NGS检测常发现多种分子异常，可与临床预后指标结合进行危险分层、指导治疗及预测耐药。目前报道的MCL重现性驱动突变基因包括TP53、ATM、CCND1、NOTCH1/2、KMT2D、NSD2、HNRNPH1、SP140等[11]。其中TP53异常［包括基因突变及（或）17p缺失］是MCL较强的预后不良指标，伴TP53异常者预后差，中位生存期仅1.8年左右；诊断时TP53突变频率为11％～25％，复发患者的TP53突变频率约为45％。TP53突变与CDKN2A缺失、KMT2D突变等常同时存在，同时存在者预后不良。推荐老年MCL完善TP53检测（基因突变及FISH检测），经济条件允许时推荐进一步完善免疫球蛋白重链可变区（IGHV）突变、FISH检测CDKN2A缺失、MYC异常等。通过NGS监测微小残留病（MRD）目前已在很多临床试验中开展并用于指导治疗。

三、诊断和鉴别诊断

老年MCL的诊断主要依据典型的组织形态学特征、免疫表型（CD5^+^、CD20^+^、CD23⁻、CyclinD1^+^、CD10^±^）和（或）t（11:14）/CCND1异常。

WHO分型包括：①经典型MCL：来源于生发中心前的B细胞，通常不伴IGHV突变，SOX11阳性。②白血病性非结节型MCL：多数临床呈惰性表现，常侵犯外周血、骨髓和脾脏，伴IGHV突变，不表达或低表达SOX11，Ki-67增殖指数通常<10％。但如出现TP53异常，可迅速进展、表现为侵袭性特征。③原位套细胞肿瘤（ISMCN）：指Cyclin D1阳性（常伴CCND1基因重排）的B细胞局限分布于淋巴滤泡套区，并未达到MCL诊断标准。ISMCN常偶然被发现，很少出现进展[12]。

经典型MCL最常见，约占全部MCL的85％，其中部分患者疾病进展快，预后极差，定义为高危的MCL，包括组织学异常（母细胞型和多形性）、临床高危（MIPI高危）和分子生物学高危。

鉴别诊断主要与其他B淋巴细胞增殖性疾病进行鉴别，尤其是慢性淋巴细胞白血病（CLL）。

MCL分期：经典型主要参考2014版Lugano分期标准，分为Ⅰ期、Ⅱ期、Ⅱ期伴大肿块和Ⅲ期、Ⅳ期；白血病性非淋巴结型MCL尚无统一分期标准，可参考CLL相关标准[12]。

MIPI：纳入指标包括年龄、ECOG评分、LDH/正常值、WBC。结合Ki-67增殖指数（阳性标准为≥30％）的MIPI-c也是目前常用的临床评分系统。

四、MCL的治疗

本专家共识推荐的治疗方案根据牛津证据分级系统（Oxford Center for Evidence-Based Medicine，OCEBM）对证据质量和推荐强度进行分级（表2）。对于部分无证据支持的临床问题，本共识依据专家临床经验，形成基于专家共识的推荐意见，即良好实践主张（Good Practice Statement，GPS）。

**表2:** 2009版牛津证据分级系统（OCEBM）的推荐及证据强度分级

推荐强度	证据强度	描述
A	1a	同质性好的多项随机对照试验（RCT）的系统评价
	1b	单个RCT研究（置信区间小）
	1c	显示“全或无”效应的任何证据
B	2a	同质性好的队列研究的系统评价
	2b	单个队列研究（包括低质量RCT，如失访率>20％）
	2c	基于患者结局的研究
	3a	同质性好的病例对照研究的系统评价
	3b	单个病例对照研究
C	4	病例系列报道（包括低质量队列研究及病例对照研究）
D	5	专家意见或评论

（一）一线治疗

老年MCL缺乏标准一线治疗方案，本共识建议根据患者危险分层及CGA体系综合评估，制定治疗策略。高危患者目前尚无统一定义，部分研究中的高危患者定义包括：①MIPI或MIPI-c评分高危组；②大包块（直径≥5 cm）；③母细胞型/多形性MCL；④Ki-67增殖指数≥30％或50％；⑤TP53突变及（或）缺失。

Ⅰ、Ⅱ期不伴高危因素患者一线治疗建议免疫化疗联合或不联合局部放疗。Ⅲ、Ⅳ期或伴高危因素的Ⅰ、Ⅱ期老年MCL患者的治疗流程见图1。同时老年患者更需重视营养支持、感染预防、粒细胞集落刺激因子等支持治疗。

**图1:**
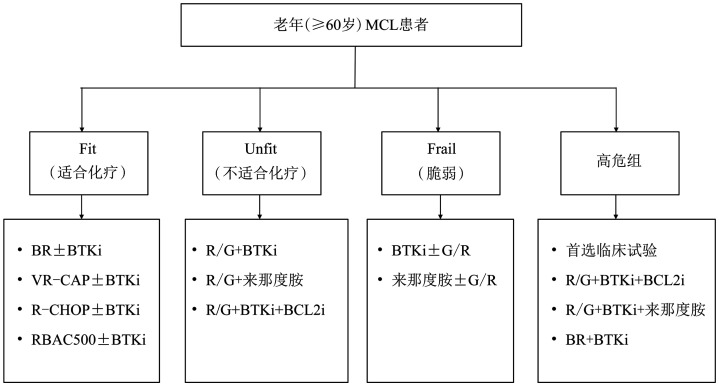
初治老年套细胞淋巴瘤（MCL）的治疗流程 **注** BR：苯达莫司汀+利妥昔单抗；BTKi：布鲁顿酪氨酸激酶抑制剂；VR-CAP：利妥昔单抗+硼替佐米+环磷酰胺+多柔比星+泼尼松；R-CHOP：利妥昔单抗+环磷酰胺+多柔比星+长春新碱+泼尼松；RBAC500：利妥昔单抗+苯达莫司汀+阿糖胞苷；R：利妥昔单抗；G：奥妥珠单抗；BCL2i：B细胞淋巴瘤/白血病-2蛋白抑制剂

利妥昔单抗（R）是MCL治疗的基石性药物，有大量循证证据，奥妥珠单抗作为新一代抗CD20单抗，在MCL中可诱发更强的细胞杀伤力。一项研究纳入LyMa和LyMa-101研究中的奥妥珠单抗和R队列，采用倾向评分匹配方法平衡两组患者的基线差异，比较两组患者的疗效和安全性，分析显示，奥妥珠单抗组较R组有更高的MRD阴性率及生存获益，但输注不良反应更高（3～4级发生率：4.7％对0.7％）[13]。本共识推荐奥妥珠单抗作为抗CD20单抗的选择之一，但应注意输注不良反应的管理（推荐强度B）。

1. 经典MCL：

（1）适合化疗的老年MCL患者：免疫化疗是此类患者的首选，缺乏标准治疗方案，BR（苯达莫司汀+利妥昔单抗）方案可作为老年MCL的优选治疗方案之一[14]–[15]。此外，R-CHOP（利妥昔单抗+环磷酰胺+多柔比星+长春新碱+泼尼松）、VR-CAP（利妥昔单抗+硼替佐米+环磷酰胺+多柔比星+泼尼松）方案可作为次选方案，治疗过程中需注意老年患者应用蒽环类药物的心脏毒性[16]–[18]。

①BR+BTKi方案：SHINE及ECHO研究均显示，BR方案联合BTKi的无进展生存（PFS）显著优于BR方案；安全性方面，总体3～4级不良事件（AE）相似，但伊布替尼联合BR组出血、房颤等发生率较BR组升高，而阿可替尼联合BR组与BR组无明显差异[9]–[10]。因此本共识建议将新一代BTKi（阿可替尼、泽布替尼和奥布替尼）联合BR方案作为老年MCL患者的优选治疗方案。BTKi使用期间应关注出血事件、心血管事件、血液学不良反应、感染等AE的管理[19]（推荐强度A）。

②RBAC500方案：BR方案联合低剂量阿糖胞苷（RBAC500）的一项Ⅱ期临床试验中，年龄>65岁且根据CGA被归类为适合化疗（fit）患者或60～65岁且不适合接受大剂量阿糖胞苷序贯自体造血干细胞移植的57例患者的完全缓解（CR）率为91％，2年PFS率和总生存（OS）率分别为81％和86％[20]，7年PFS率和OS率分别为55％和63％[21]。此方案长期疗效优异且无需R维持，但安全性需密切关注，最常见的3～4级AE包括血小板减少症和中性粒细胞减少症，72％的患者需要降低剂量。既往其他研究显示，RBAC500减量或予2 d阿糖胞苷（100～500 mg/m^2^，第2～3天），毒性显著降低且疗效仍可维持[20],[22]。因此，建议临床应用RBAC500方案的过程中根据血液学不良反应适当调整剂量，加强支持治疗（推荐强度B）。

苯达莫司汀对T淋巴细胞的功能和水平有显著抑制作用，应特别警惕感染发生[23]。同时，有研究显示，其对CAR-T细胞的疗效可能产生负面影响，洗脱时间应大于9个月[24]。对有潜在CAR-T细胞治疗需求的患者，应尽量避免短期内应用苯达莫司汀（推荐强度D）。

（2）不适合化疗的老年患者：应考虑疗效和安全性之间的平衡，“无化疗”方案可作为这类人群的首选，可选择的治疗方案如下：

①BTKi+R方案：ENRICH研究比较了伊布替尼联合利妥昔单抗（IR方案）与常规化疗（R-CHOP或BR方案）序贯R维持治疗的疗效及安全性，结果显示，IR组的PFS显著优于常规化疗组，两组的中位PFS期分别为65.3个月和42.4个月，其中IR方案与R-CHOP方案比较，PFS获益更明显。安全性方面，三组3级以上AE发生率类似，但IR组3级以上中性粒细胞减少的发生率明显低于化疗组，且IR组患者生活质量提升更显著，但IR组≥3级房颤发生率更高（6.1％对0.5％）[25]。AR方案（阿可替尼联合利妥昔单抗）治疗老年初治MCL的Ⅱ期研究显示，CR率为90％，2年PFS率和OS率分别为92％和96％，3级以上AE发生率<1％[26]。ALTAMIRA研究是另一项AR方案治疗初治老年MCL患者的Ⅱ期研究，纳入部分高危患者，结果显示，高危组（如母细胞型/多形性、TP53突变或Ki-67增殖指数>30％）患者应用AR方案的疗效及预后欠佳[27]。

基于上述研究，推荐BTKi联合R作为非高危、老年、不适合化疗患者的优选治疗（推荐强度A）。

②R^2^方案（来那度胺联合利妥昔单抗）：R^2^无化疗方案在初治MCL中的疗效和安全性研究显示，R^2^方案可实现较高缓解率，总有效率（ORR）为92％，CR率为64％[28]。最新的长期随访结果显示，R^2^方案9年PFS率和OS率分别为51％和66％[29]。安全性方面，诱导期3～4级血液学AE主要包括中性粒细胞减少（42％）、血小板减少（13％）和贫血（8％）；3～4级非血液学AE主要为皮疹（29％）。维持治疗过程中3～4级血液学AE主要包括中性粒细胞减少（42％）、血小板减少（5％）和贫血（3％），且21％的患者发生继发性恶性肿瘤，5％为侵袭性肿瘤，其余为局部治疗的非侵袭性皮肤癌。基于此，本共识推荐R^2^方案作为治疗方案用于不适合化疗的老年患者（推荐强度B）。

③BTKi+R+BCL-2抑制剂（BCL2i）方案：OASIS Ⅱ研究证实，患者接受IR方案联合BCL2i维奈克拉治疗6个周期后MRD阴性率由IR方案的53.8％提升至82.1％，缓解深度加深。但同时不良反应发生率显著增加，IR方案联合维奈克拉组≥3级中性粒细胞减少症发生率较IR方案组显著升高（34％对11.8％），3级及以上AE发生率更高（64％对47.1％）[30]。基于此，BTKi+R+BCL2i也可作为不适合化疗尤其是高危组老年患者的治疗方案（推荐强度B）。

（3）脆弱患者：脆弱的老年MCL患者可能会经历更多的治疗相关不良反应和更差的预后。在许多情况下，可以优先考虑患者的生活质量和缓解症状，而不是缓解深度。对于计划接受全身治疗的患者，应考虑在疾病肿瘤负荷导致功能受损的情况下先使用激素，请老年病学专家参与并优化合并症诊疗。新型靶向药物和无化疗方案已成为老年脆弱患者治疗探索的方向。BTKi±R、来那度胺±R可作为此类人群的治疗选择（推荐强度D）。

2. 高危患者：首选推荐是参加设计良好的临床试验。免疫化疗虽然可在一定程度上延长患者的生存期，但患者总体预后较差，以靶向治疗（如BTKi、BCL2i、来那度胺）为基础的联合治疗可作为此类患者的重要选择。

TP53异常是MCL公认预后最差的生物学因素，BOVEN研究探索了泽布替尼+维奈克拉+奥妥珠单抗在此高危人群中的应用，共纳入25例TP53异常的初治MCL患者，ORR和CR率分别为96％和88％，治疗13个周期时MRD阴性率高达95％，中位随访28.2个月，2年PFS率和OS率分别为72％和76％，3年PFS率、OS率和疾病特异性生存率分别为64％、76％和95％[31]；阿可替尼+奥妥珠单抗+维奈克拉也在TP53异常人群中获得与BOVEN研究一致的疗效[32]。基于此推荐，BTKi+BCL2i+奥妥珠单抗可作为TP53异常MCL的优选治疗方案（推荐强度B）。

在亚组人群中分析了其他无化疗方案如BTKi+来那度胺+利妥昔单抗在TP53异常患者中的疗效，CR率和ORR分别为64％和73％[33]–[34]。Polaris研究显示，奥布替尼、来那度胺联合利妥昔单抗的最佳ORR和CR率分别为100％和89.7％，24个月PFS率为85.9％[35]。基于此，推荐BTKi+来那度胺+利妥昔单抗作为此类患者的治疗选择之一（推荐强度B）。

BTKi联合BR方案在部分高危人群中显示出了一定疗效，ECHO研究的亚组分析显示，与BR方案组比较，阿可替尼联合BR方案组在Ki-67增殖指数≥30％（*HR*＝0.55）和大包块（直径≥5 cm）（*HR*＝0.63）患者中获益显著，而其他高危患者包括母细胞型/多形性，TP53突变或MIPI高危显示出一定获益趋势[10]。基于此，BTKi联合BR方案可作为高危人群的次选方案（如仅具有Ki-67增殖指数≥30％或大包块高危特征，可作为优选方案）（推荐强度B）。

（二）维持治疗

1. 利妥昔单抗维持治疗：多项研究显示，老年MCL患者接受R-CHOP方案治疗缓解后应用R维持治疗，PFS获益，疾病进展或死亡的风险可降低[16],[36]。因此，本共识推荐R-CHOP方案治疗缓解后，予R维持治疗（375 mg/m^2^，每2～3个月1次，维持2～3年或至疾病进展）（推荐强度A）。

BR方案治疗缓解后是否需要R维持存在一定争议，MAINTAIN研究未显示R维持治疗能使PFS或OS获益[37]。部分回顾性研究支持应用BR方案后使用R维持治疗[38]–[40]，近期的大型真实世界队列分析提示，患者应用BR方案后接受R维持治疗能够延长至下一次治疗时间（TTNT）及OS期[38]。且多项老年MCL的Ⅲ期临床研究中BR方案治疗组均接受了R维持治疗[9]–[10],[25]。因此，BR方案治疗后可考虑R维持治疗，但目前尚未在前瞻性研究中确定其获益，是否应用R维持治疗应个体化考虑（推荐强度B）。

2. BTKi维持治疗：BTKi维持治疗缺乏前瞻性大型临床研究，且维持治疗时间尚不统一。一项Ⅱ期多中心研究在诱导治疗后达到CR或部分缓解的MCL患者中予固定持续时间为4年的伊布替尼维持治疗（560 mg/d），3年PFS率和OS率分别为94％和97％，年龄≥65岁老年MCL与年轻患者的预后无差异，最常见不良反应为感染（发生率为86％，1～2级为主）（推荐强度B）[41]。

3. 双药维持治疗：SHINE及ECHO研究应用R+BTKi双药维持治疗，BTKi持续应用至疾病进展，R 375 mg/m^2^，每8周1次，持续2年（推荐强度B）[9]–[10]。

（三）复发/难治（R/R）老年MCL的治疗

对于R/R MCL患者，建议在病情允许的情况下再次进行病理活检，以明确复发情况并评估是否存在高危亚型（母细胞型或多形性）。若经济条件允许，建议完善NGS检查，以评估复发后的高危因素。

治疗方面，首选参加临床研究，无临床研究时可根据患者前期治疗方案、高危因素及CGA进行选择。高危因素包括：①TP53突变或缺失；②母细胞型/多形性MCL；③Ki-67增殖指数≥50％。

既往未接受BTKi治疗的患者首选BTKi治疗。接受过BTKi连续治疗的患者，传统的化疗方案疗效有限，但前期研究显示，RBAC500方案治疗有效率达83％，CR率为60％，可作为适合化疗患者的治疗选择[42]；同时，新型靶向药物及细胞免疫治疗（如非共价BTKi、来那度胺、BCL2i、CAR-T细胞和双特异性抗体等）单独或联合应用为R/R MCL患者带来更多希望，可作为优选治疗。

1. BTKi+BCL2i：BTKi单药在R/R MCL中的疗效仍有待提升，联合治疗是重要的治疗策略，BTKi与BCL2i联合可以提高缓解率，延长PFS期。Ⅲ期SYMPATICO研究探索维奈克拉联合伊布替尼在R/R MCL中的疗效及安全性，结果显示，联合治疗组的CR率和PFS期较伊布替尼单药组显著改善（CR率：54％对32％，中位PFS期：31.9个月对22.1个月）。安全性方面，两组严重AE发生率一致，包括中性粒细胞减少症、肺炎和血小板减少症，≥3级房颤发生率均为5％[43]。进一步汇总SYMPATICO研究中74例TP53突变MCL患者接受伊布替尼联合维奈克拉治疗的疗效和预后，结果显示，TP53突变的MCL患者接受联合治疗具有较高的缓解率和较长的生存期，CR率可达57％，中位缓解持续时间（DoR）为26个月，中位PFS期为20.9个月（推荐强度B）[44]。

2. 非共价BTKi：匹妥布替尼是首个获批用于R/R MCL的非共价BTKi[45]–[46]。匹妥布替尼单药治疗的Ⅰ/Ⅱ期研究中位随访12个月，既往未应用BTKi患者（14例）的ORR和CR率分别为85.7％和35.7％，中位DoR和PFS期均未达到。BTKi经治患者（90例）的ORR和CR率分别为57.8％和20％，中位DoR和PFS期分别为21.6和7.4个月；其中入组前因疾病进展停止接受共价BTKi治疗患者（74例）的ORR为50％，中位DoR和PFS期分别为14.8个月和5.5个月。患者整体耐受性良好，3级及以上出血和房颤发生率较低，分别为4％和1％，且仅有3％患者因治疗相关AE停药。同时，研究显示，在因心脏不良反应停用共价BTKi治疗的患者中，75％应用匹妥布替尼未再发心脏AE[47]。因此，匹妥布替尼对老年脆弱及共价BTKi不耐受患者提供了一种不良反应更少的治疗选择。尽管联合治疗尚无数据，基于上述结果，联合治疗可能带来更大的疗效获益，应视为更优的选择（推荐强度B）。

3. CAR-T细胞：Brexu-cel（KTE-X19）已获批用于R/R MCL患者的治疗。ZUMA-2研究纳入32例年龄超过65岁患者，中位随访35.6个月，ORR和CR率分别为91％和68％；中位DoR、PFS期和OS期分别为28.2、25.8和46.6个月。上述结果表明，CD19 CAR-T细胞能够使老年患者持久缓解，不良反应可耐受，高危患者亦有获益趋势[48]。另一项来自国际血液和骨髓移植研究中心（CIBMTR）的前瞻性非干预性研究共纳入456例患者，其中42％的患者有TP53/17p缺失和（或）Ki-67指数≥50％等高危因素，高危亚组的ORR和CR率与总体患者一致。因此推荐一线复发后伴高危因素或二线治疗未达CR或BTKi治疗失败患者尽早考虑CD19 CAR-T细胞治疗[49]（推荐强度B）。

4. 双特异性抗体：在一项Ⅰ/Ⅱ期研究中，既往接受过多线治疗的MCL患者应用固定疗程的双特异性抗体格菲妥单抗（CD20/CD3双特异性抗体）单药治疗获得了深度且持久的缓解，且安全性可控。研究共纳入60例患者，中位年龄72岁，随访17.2个月，CR率为78.3％，ORR为85.0％。在既往未接受BTKi治疗和接受过BTKi治疗的患者中均有较高的CR率（86.2％和71.0％）和ORR（96.6％和74.2％）。患者接受固定疗程的格菲妥单抗单药治疗显示出持续的CR，数据截止时，大多数CR患者（59.6％）仍处于缓解状态，所有患者的中位DoR为16.2个月。且安全性可控，细胞因子释放综合征主要为1～2级（推荐强度B）[50]。

脆弱患者可尝试应用BTKi、来那度胺、BCL2i，双特异性抗体等单药或联合治疗，以改善症状为主（推荐强度D）。

五、疗效评价及随访

MCL的疗效评价标准参照Lugano 2014标准进行，同时MCL常侵犯外周血或骨髓，有条件单位可考虑进行MRD监测。

完成治疗后的前2年应每3个月随访1次，完成治疗后3～5年每半年随访1次，5年后每年随访1次或有症状时进行检查，如有异常或考虑疾病进展，则及时完善检查。
